# The mechanism and risk factors for immune checkpoint inhibitor pneumonitis in non-small cell lung cancer patients

**DOI:** 10.20892/j.issn.2095-3941.2020.0102

**Published:** 2020-08-15

**Authors:** Xiaoyang Zhai, Jian Zhang, Yaru Tian, Ji Li, Wang Jing, Hongbo Guo, Hui Zhu

**Affiliations:** ^1^Department of Radiation Oncology, Shandong Cancer Hospital and Institute, Shandong First Medical University and Shandong Academy of Medical Sciences, Jinan 250117, China; ^2^Department of Thoracic Surgery, Shandong Cancer Hospital and Institute, Shandong First Medical University and Shandong Academy of Medical Sciences, Jinan 250117, China; ^3^Department of Radiation Oncology, Shandong Cancer Hospital and Institute affiliated with Shandong University, Jinan 250012, China

**Keywords:** Immune checkpoint inhibitor, non-small-cell lung cancer, pneumonitis, risk factors

## Abstract

Immune checkpoint inhibitors (ICIs) are new and promising therapeutic agents for non-small cell lung cancer (NSCLC). However, along with demonstrating remarkable efficacy, ICIs can also trigger immune-related adverse events. Checkpoint inhibitor pneumonitis (CIP) has been reported to have a morbidity rate of 3% to 5% and a mortality rate of 10% to 17%. Moreover, the incidence of CIP in NSCLC is higher than that in other tumor types, reaching 7% to 13%. With the increased use of ICIs in NSCLC, CIP has drawn extensive attention from oncologists and cancer researchers. Identifying high risk factors for CIP and the potential mechanism of CIP are key points in preventing and monitoring serious adverse events. In this review, the results of our analysis and summary of previous studies suggested that the risk factors for CIP may include previous lung disease, prior thoracic irradiation, and combinations with other drugs. Our review also explored potential mechanisms closely related to CIP, including increased T cell activity against associated antigens in tumor and normal tissues, preexisting autoantibodies, and inflammatory cytokines.

## Introduction

Lung cancer has the highest morbidity and mortality rates of malignant tumors, with over 2.09 million diagnosed cases and 1.76 million deaths estimated in 2018^1,2^. Non-small cell lung cancer (NSCLC) is the most common histological type and has a poor prognosis because most patients are diagnosed at an advanced stage^3–6^. The 5-year survival rate for advanced NSCLC is only 2.8%^7^. Immune checkpoint inhibitors (ICIs) are promising new immunotherapeutic drugs that reactivate T cells to kill tumor cells by the blocking programmed cell death protein 1/ligand 1 (PD-1/PD-L1) pathway or the cytotoxic T lymphocyte-associated antigen 4 (CTLA-4) pathway. A growing number of studies have indicated that ICIs can improve the clinical outcomes of advanced NSCLC^8,9^. ICIs can achieve a 5-year overall survival (OS) rate of 23.2% when used as first-line therapy. For patients with PD-L1 tumor proportion score (PD-L1-positive tumor cells/total number of viable tumor cells) of 50% or greater, the 5-year OS rate can reach 29.6%^10^. Based on these results, ICIs have been approved as first-line therapies for advanced NSCLC by the US Food and Drug Administration (FDA). However, along with providing an excellent survival benefit, ICIs can also induce specific hyperactivation of the immune response, resulting in normal tissue damage^11,12^. Immune-related adverse events (irAEs), such as rash, colitis, hepatitis, myocarditis, endocrinopathies, and pneumonitis, are commonly reported^13^. Pneumonitis, termed checkpoint inhibitor pneumonitis (CIP), is particularly worrisome among the irAEs^14–16^. Although the incidence of CIP has been reported to be 3% to 5%, it has a fatality rate of 10% to 17%. However, in NSCLC, the morbidity of CIP is 7% to 13%^15,17–22^. CIP occurs mainly in the first 6 months after treatment^14^. The major symptoms are dyspnea, cough, fever, and chest pain^23^. High-resolution computed tomography is the preferred diagnostic method when CIP is suspected^24,25^. The traditional treatment for CIP is corticosteroid administration. Additional immunosuppressive agents are necessary for steroid-refractory pneumonitis^13^. However, current studies on CIP have mainly focused on the incidence, diagnosis, and management of this adverse event. Studies concerning the risk factors for CIP are limited. In this study, we reviewed the potential mechanisms and risk factors for CIP in NSCLC patients with the aim of identifying patients with a high probability of CIP, to ensure close monitoring during the course of immunotherapy treatments.

## Mechanism of CIP

**Figure 1:**
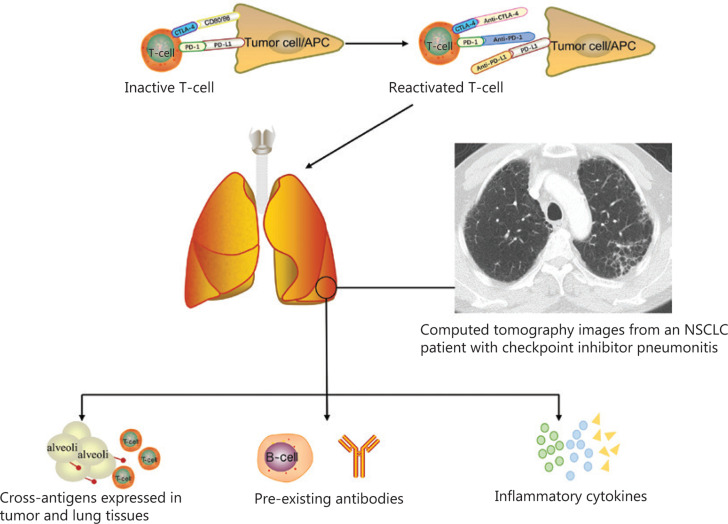
The potential mechanism for checkpoint inhibitor pneumonitis.

The mechanism of CIP remains unclear, but it is believed to be related to the immune dysregulation caused by ICIs. Postow et al.^26^ suggested four potential mechanisms underlying irAEs. First, the occurrence of adverse events may be related to increased T cell activity against cross-antigens expressed in tumor and normal tissues. Suresh et al.^27^ found that bronchoalveolar lavage (BAL) samples from CIP patients exhibited increased lymphocytosis, mainly composed of CD4+ T cells. Importantly, the authors observed increased central memory T cell (Tcm) numbers and decreased CTLA-4 and PD-1 expressions within the Treg population. PD-1^+^ and CTLA-4^+^ Tregs have negative regulatory effects on CD8+ T cells, conventional T cells (such as Tcms), and macrophage proinflammatory responses^28,29^. Therefore, increasing activated alveolar T cell numbers and attenuating the anti-inflammatory Treg phenotype may lead to dysregulation of T cell activity. In the tumor microenvironment, reactivated tumor infiltrating lymphocytes (TILs) have the potential to provide an accurate prognosis for NSCLC patients^30^. A meta-analysis of 8,600 patients with lung cancer indicated that a high level of CD8+ T cell infiltration in the tumor nest and tumor stroma, and CD4+ T cell infiltration in the tumor stroma showed better survival. Conversely, a high level of FOXP3+ Tregs in the tumor stroma was related to poor outcomes^31^. Another meta-analysis of NSCLC obtained similar results and showed that TILs had a predictive role for OS and recurrence^32^. Although lymphocytosis was observed in BAL samples from CIP patients, its predictive value of CIP lacks sufficient evidence, and the potential relationship between these parameters needs future exploration. Second, increased levels of preexisting autoantibodies may also be responsible for irAEs. Recent studies have shown that preexisting anti-rheumatoid factor antibodies, antinuclear antibodies, anti-thyroglobulin antibodies, and anti-thyroid peroxidase antibodies are potentially related to the development of irAEs in NSCLC patients^33^. However, unlike the predictive role of preexisting rheumatoid factor in skin reactions and preexisting anti-thyroid antibodies in thyroid dysfunction, the specific antibodies related to CIP are still being explored. Third, increases in the levels of inflammatory cytokines are also related to the appearance of irAEs. An NSCLC patient who developed CIP after atezolizumab treatment was reported to have elevated levels of C-reactive protein and interleukin-6 (IL-6), when compared with baseline levels^34^. Cytokines can also serve as biomarkers for adverse events, and their elevated expression correlates with severe ICI toxicity^35,36^. The fourth possible mechanism is that anti-CTLA-4 antibodies can directly bind with CTLA-4 expressed on normal tissues, such as the pituitary gland. This mechanism may also be the reason why pituitary inflammation is a specific adverse event of anti-CTLA-4 antibodies^37,38^. According to the results of these studies, we speculate that the first three mechanisms may be the major causes of CIP, which are summarized in **Figure 1**. Additional potential mechanisms still require further exploration and verification.

## Risk factors for CIP

To date, the incidence of CIP is approximately 5% for any grade and 1% for grade 3 or higher pneumonitis in patients treated with anti-PD-1/PD-L1 antibodies^23^. With the widespread application of ICIs, the incidence is expected to increase. A study showed that prior lung disease, prior thoracic radiotherapy, and prior combination therapy were significant risk factors for pneumonitis (odds ratios: 2.86, 3.34, and 2.73, respectively)^39^. Moreover, other potential risk factors for CIP include previous or current smoking, an age older than 70 years, PD-1 inhibitor treatment, and histological type. Reviews on risk factors for CIP will facilitate an early diagnosis and management of high risk groups. The detailed risk factors are listed in **Table 1**.

### Previous lung disease

Previous lung diseases associated with CIP may include chronic obstructive pulmonary disease (COPD), asthma, interstitial lung disease (ILD), pulmonary fibrosis, pneumothorax, and pleural effusion. The incidences of CIP in patients with COPD and asthma were reported to be 2.3% higher than that in patients without COPD and asthma^40^. Additionally, a history of smoking may augment the pneumonitis incidence in asthma patients^41^. The leading reasons may be the decline in pulmonary function and poor resistance to outside factors in COPD and asthma patients. However, recent studies have reported that the numbers of CD4+ cells with PD-1 expression increased in COPD patients^42–44^. Therefore, patients with mild COPD may have a higher morbidity of CIP but a longer progression-free survival (PFS) than those without COPD when using ICIs. In a retrospective study enrolling 216 NSCLC patients receiving nivolumab, the morbidity of patients with preexisting ILD was significantly higher than that of patients without ILD (31% *vs.* 12%; *P* = 0.014)^45^. Interstitial pulmonary fibrosis is a pathogenicity of ILD. A retrospective analysis showed that preexisting pulmonary fibrosis was closely associated with the risk of anti-PD-1 antibody-related pneumonitis^46^. The development of pneumonitis was shown to be related to preexisting lung disease, in a study including pulmonary fibrosis, pneumothorax, and pleural effusion^39^. Patients with CIP treated with steroids exhibited a high remission rate and low mortality rate^47,48^. Therefore, a history of previous lung diseases is not a contraindication for immunotherapy. However, clinicians should be vigilant about CIP when ICIs are used in patients with previous lung disease.

### Thoracic radiotherapy (RT)

The current model for cancer treatment is increasingly inclined towards multidisciplinary comprehensive treatments. RT is widely used in the treatment of primary tumors and metastatic lesions. Additionally, this treatment can reduce disease recurrence and improve OS in patients with multiple tumors^49–51^. In addition, studies have reported a synergistic effect between RT and immunotherapy^52,53^. However, the incidence of pneumonitis in patients with prior radiotherapy may be elevated because of damaged pulmonary function caused by thoracic irradiation.

Several studies have suggested that a history of previous RT is a potential risk factor for CIP. The Keynote-001 trial investigated the efficacy of pembrolizumab in NSCLC patients^54^. Secondary analysis of the trial compared PFS, OS, and pulmonary toxicity between patients who received thoracic RT before immunotherapy and those who did not. The patients who received thoracic RT (*n =* 24) before pembrolizumab administration had a higher incidence of any-grade pulmonary toxicity than those who did not receive thoracic RT (13% *vs.* 1%; *P =* 0.046); however, no significant difference in the risk of grade ≥ 3 pneumonitis was observed between the two groups (4% *vs.* 1%, with only one event observed in each group; *P =* 0.44)^55^. However, the median interval time between thoracic RT and pembrolizumab administration was 11.5 months; thus, whether a shorter interval between ICI treatment and thoracic RT can increase the risk of toxicity remains unknown. A new model of immunotherapy being administered concurrently with chemoradiotherapy is also being assessed in the DETERRED and PACIFIC-2 trials^56^. The rate of grade 2 or higher pneumonitis was 10% in the DETERRED trial^57^. It appears that concurrent chemoradiotherapy did not significantly increase toxicity when compared with immunotherapy after RT, but further follow-up is needed.

A recent retrospective analysis of 188 NSCLC patients showed that RT parameters (technique, timing, courses, and prior chest-RT dosimetric parameters) were not associated with immune-related pneumonitis. Notably, a study showed that the incidence of pneumonitis was higher in an RT group with curative intent than in an RT group with palliative intent (89% *vs.* 11%; *P* = 0.051)^58^. Moreover, the timing of RT is very important, and adding RT before or after immunotherapy remains controversial^59^. A retrospective review recently found that RT following immunotherapy was relevant to improved survival. However, this finding could be explained by the relatively good general status and the reduced progression in patients treated with RT after immunotherapy^60^.

It is difficult for clinicians to differentiate whether the cause of pneumonitis is related to radiation or immunotherapy. Radiation pneumonitis (RP), an early change in radiation-induced lung injury, usually occurs between 1 and 3 months after RT^61^. The median time of CIP onset is 82 days after immunotherapy, which is similar to that of RP^14^. The main imaging features of the two types of pneumonitis are ground glass opacity or diffuse haziness, and the pathological feature is lymphocytic alveolitis^23,61–63^. However, RP mostly occurs in the radioactive field, and CIP is mainly found within the low dose range or outside the RT fall-off dose region^58^. Notably, although it is difficult to distinguish the two types of pneumonitis in patients previously treated with both treatments, the first-line therapy for both is corticosteroids^25,61^. Radiomics, an emerging field, provides a new method to predict immunotherapy-induced pneumonitis. This technique automatically extracts imaging features from medical imaging data for analysis by synthesis^64^. A related radiomics trial is ongoing (NCT03305380), which may offer a new approach for the diagnosis and prediction of CIP.

### Combination with ICIs or other drugs

ICIs are usually combined with chemotherapeutic drugs, tyrosine kinase inhibitors, or additional immune-targeted drugs. In a meta-analysis of 4,496 patients, the incidence of pneumonitis in patients treated with a PD-1 inhibitor or combination therapy was 2.7% *vs.* 6.6%, respectively^20^. The combination of ICIs and chemotherapy, as a recommended treatment according to guidelines, is increasingly used in the clinic. Pembrolizumab combined with chemotherapy prolongs the median OS of NSCLC patients by nearly 5 months. However, the incidence of any-grade CIP was found to be increased threefold in a combination group compared with a chemotherapy group^65^. Notably, in the preclinical phase of the Ib TATTON study, the reported incidence was 2.9% for osimertinib monotherapy and 38% for osimertinib combined with durvalumab^66^. Treatment in the concurrent treatment group was paused because of high rates of pneumonitis. Oshima et al.^67^ also evaluated the incidence of pneumonitis in patients treated with epidermal growth factor receptor (EGFR) tyrosine kinase inhibitors (EGFR-TKIs) with or without nivolumab, which produced incidence rates of 25.7% and 4.59%, respectively. Thirteen of 18 cases of pneumonitis treated with both EGFR-TKIs and nivolumab developed after the discontinuation of nivolumab. The average interval time was 71.1 days. In addition to being related to the order of treatment, pneumonitis may be associated with specific TKIs. One recent study showed that the sequential use of PD-(L)1 inhibitors followed by osimertinib within 3 months resulted in more severe irAEs than treatment with osimertinib followed by PD-(L)1 inhibitors or other TKIs following PD-(L)1 blockade^68^. However, the incidence of pneumonitis following osimertinib monotherapy is higher than that following gefitinib or erlotinib therapy, which was shown in the FLAURA study^69^. This adverse reaction may be magnified by combination treatment with ICIs. Notably, the treatment modality of osimertinib plus ICIs in clinical practice is limited. Currently, it is widely considered that PD-1/PD-L1 inhibitors have restricted efficacy in patients with mutation types. Regardless of the risk of CIP, the treatment regimen itself is also controversial. A higher incidence of CIP was observed in the double-immune checkpoint inhibitor groups than in the other control groups in the Checkmate 012, Checkmate 227, and Checkmate 568 clinical trials^70–72^. However, adverse events were tolerated, and no novel toxicities occurred with combination treatment when compared with single treatment^71^.

In conclusion, with the popular use of combination therapy, the incidence of CIP will inevitably increase. Clinicians must consider a patient’s general condition, degree of disease, times receiving treatment, and the risk of CIP. The incidence of CIP after immunotherapy combined with other drugs is summarized in **Table 2**. The mechanism underlying the increased incidence of CIP after combination therapy is not clear. The relatively long duration of treatment and relatively increased antigen or cytokine release may account for the increased incidence of adverse reactions. Further studies are still needed to evaluate the safety and high risk factors for combination therapy.

### Other risk factors

The occurrence of CIP may be related to age, smoking history, drug type, treatment history, and histological type. A previous study found that patients older than 70 years of age were more common in a CIP group than a non-CIP group (54.5% *vs.* 30.3%; *P* = 0.025)^42^. This finding can be explained by the decline in pulmonary function and increase in medical complications in the elderly population. A history of smoking is also a risk factor for CIP. Former/current smokers were found to have a higher incidence of pneumonitis than non-smokers (*P* = 0.03)^79^. The high incidence of CIP in NSCLC patients may be due to a history of smoking and a consequential decline in pulmonary function^80^. Notably, sex may also have an association with the occurrence of CIP. Suresh et al.^14^ reported that females had a higher incidence of CIP than males, but the difference was not significant. Although this result has yet to be confirmed, it does provide a direction for further study. In addition, the incidence of any-grade pneumonitis has been found to be significantly higher in patients receiving PD-1 inhibitors than in those receiving PD-L1 inhibitors (*P* = 0.001)^21^. This finding may be because anti-PD-1 drugs can affect the PD-L1 and PD-L2 pathways, while anti-PD-L1 drugs can only influence the PD-L1 pathway. However, pembrolizumab and nivolumab have shown no significant difference in causing CIP morbidity^20^. Treatment-naive patients may have a higher incidence of any-grade pneumonitis than previously treated patients (*P* = 0.03)^21^. ICIs have been approved as a first-line treatment for NSCLC; therefore, treatment history should be seriously considered. Notably, patients with squamous histology have a higher incidence but a lower mortality rate of immune-related pneumonitis than those with adenocarcinoma histology (*P <* 0.05)^8,9,14,75,76,81^. However, the phenomenon may be determined by the characteristics of the tumor histology itself rather than those of CIP.

The prevalence of patients with the abovementioned primary risk factors is shown in **Table 3**. Noteworthy, the incidence of CIP between random clinical trials and the real world was distinguishing. In clinical trials, because of strict inclusion criteria, patients with poor general condition were frequently excluded, which led to an underestimated morbidity rate of CIP. According to a previous report, the incidence of pneumonitis in the real world can reach as high as 19% and was much higher than the pneumonitis rate of 3%–5% reported in clinical trials^14^. With increasing use of ICIs and greater awareness about CIP, the rate of CIP in the real world should be higher in future studies.

## Management of CIP

**Figure 2:**
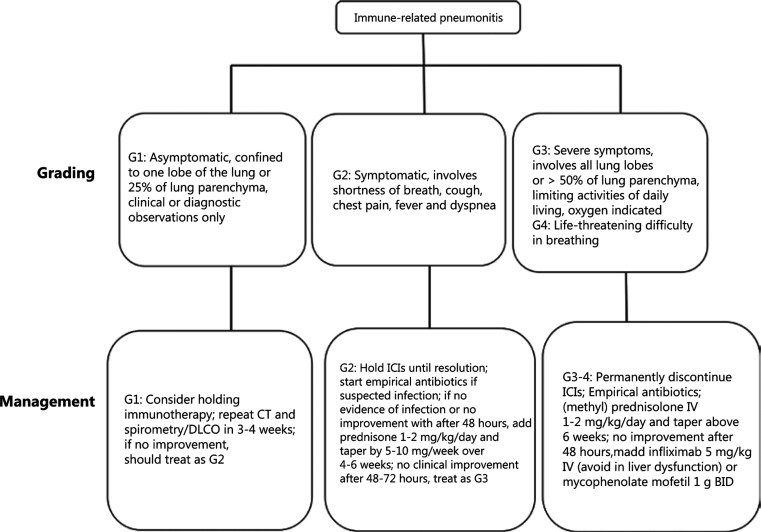
Management of checkpoint inhibitor pneumonitis. Abbreviations: G: grading; DLCO: diffusion capacity for carbon monoxide; ICIs: immune checkpoint inhibitors.

Guidelines on immunotherapy-related toxicity recommend corticosteroids as the main therapeutic modality for CIP^25,83,84^. If no remission is observed after 48 hours, immunosuppressive agents are recommended. The specific management approach is shown in **Figure 2**. Retrospective data from a large cohort study showed that 1 out of 10 patients receiving steroid therapy required additional immunosuppressive therapy^85^. However, there is still debate regarding which immunosuppressant to use. Infliximab, a monoclonal anti-tumor necrosis factor-α (TNF-α) antibody, is recommended as the first-line immunosuppressive drug for steroid-refractory CIP. Although NSCLC patients with steroid-refractory CIP have benefited from infliximab^86^, the recommendation is based on extrapolation from the efficacy of infliximab in managing immune-related colitis and lacks pathophysiological support. Notably, infliximab itself can cause interstitial pneumonitis and liver injury^87–89^. As a recommended second-line drug, mycophenolate mofetil (MMF) is still controversial as a treatment for steroid-refractory CIP. The recommendation was mainly based on its efficacy in treating immune-related hepatitis^90^. However, data from patients with ICIs have shown that MMF has negative effects on the T cell response^91^. Many cytokines, including IL-1, IL-6, and TNF-α, are continuously secreted in response to the acute inflammatory phase of CIP^91^. IL-6 and IL-1β have been reported to promote cancer progression and metastases^92,93^. Therefore, without affecting immunotherapy efficacy, IL-6 blockade (tocilizumab), IL-1 blockade (anakinra), and TNF-α blockade (infliximab) may be possible approaches to treat steroid-refractory CIP. An NSCLC patient with immune-related pneumonitis was treated with tocilizumab after the initiation of steroid therapy and showed significant symptomatic relief within 2 days of hospitalization^34^. However, the efficacy and safety of these agents still require further investigation.

## Predictive factors of CIP

In NSCLC, the prevalence of CIP is 7% to 13%. With increasing application, the incidence may increase. However, predictive factors regarding CIP are still being explored. The potential predictive factors reported to date have mainly focused on cellular biomarkers and cytokines/chemokines^94^. A retrospective study of 101 patients with melanoma indicated that increased white blood cell counts and decreased relative lymphocyte counts correlated with G3/4 lung and gastrointestinal irAEs^95^. Another study also showed that higher baseline lymphocyte counts were associated with irAEs in solid tumors^96^. In melanoma patients with severe irAEs, peripheral blood samples were evaluated at an early time point during treatment, and 11 elevated cytokines were noted, including granulocyte colony-stimulating factor, granulocyte-macrophage colony-stimulating factor, fractalkine, fibroblast growth factor 2, IFN-a2, interleukin-12p70, IL-1a, IL-1b, IL-1 receptor antagonist, IL-2, and IL-13. A predictive model composed of these 11 cytokines was verified in a validation group^36^.

## Future directions for CIP

Although ICIs have been approved as first- and second-line treatments for multiple solid tumors, many questions remain. First, the ability of biomarkers to predict irAEs is still unclear. The present biomarkers are mostly related to the mechanism of occurrence of irAEs. Clinicians can detect and manage adverse events earlier by evaluating these biomarkers. Second, the relationships between the development of CIP and tumor response or OS remain controversial. Most studies have indicated that NSCLC patients with irAEs have an improved prognosis^97–99^. However, there is now emerging evidence that the development of CIP, unlike that of other irAEs, is associated with decreased treatment efficacy and survival in ICI-treated NSCLC patients. Notably, the results regarding this topic were all from retrospective analyses, which were inevitably influenced by bias even after statistical adjustment. Therefore, this issue still needs evaluation in prospective and large sample studies^100–102^. Third, the morbidity and mortality of CIP in diverse NSCLC histological types are confusing. Differences may be related to the intrinsic characteristics of tumor histological types.

## Radiation recall pneumonitis after immunotherapy should be considered

Radiation recall pneumonitis (RRP) is acute inflammation triggered by certain pharmacological agents in previously irradiated areas^103^. The mechanisms of RRP are still unclear. Potential hypotheses involve damage to stem cells in the irradiated area and hypersensitivity of renewed cells^104,105^. Cases of RRP have been reported after chemotherapy and targeted therapy^106–108^. The incidence of EGFR-related RRP is 4.4%, yet the incidence of RRP in patients who received targeted therapy within 90 days after radiotherapy was tenfold higher than that in patients who received targeted therapy more than 90 days after radiotherapy^106^. The median time interval between the end of RT and the initiation of RRP induced by cytotoxic drugs or TKI drugs was 95 days and 124 days, respectively, as previously reported^106,107^. Immunotherapy, as an emerging therapeutic option, can also induce RRP. Two patients treated with nivolumab were reported to suffer from RRP within two years of radiotherapy completion, which is different from the windows for chemotherapy and targeted therapy^109^. RRP triggered by pembrolizumab has also been detailed in one case report^110^. Oncologists should be alert for RRP when radiological findings occur in irradiated areas following the application of drugs. Based on clinical experience, RRP is currently considered to be sensitive to steroids. The model of RT plus immunotherapy will be increasingly used in the clinic based on the PACIFIC study, and RRP needs to be given more attention. Moreover, distant toxicity induced by RT after immunotherapy is also noteworthy. The abscopal effect from immunotherapy combination with radiotherapy refers to tumor regression in a non-irradiated site^111^. One SCLC patient received peripancreatic radiotherapy after nivolumab and developed bilateral CIP^112^, which suggested that the mechanism related to the abscopal effect may also trigger immune-related adverse events in nonirradiated sites.

## Conclusions

CIP is an immune-related adverse event with relatively low morbidity and high mortality, which is relatively common in NSCLC patients. With the extensive use of ICIs in NSCLC, CIP has attracted widespread attention. Although the mechanism of CIP is still unclear, it is certain that immune dysfunction plays an important role in the development of irAEs. The risk factors for CIP are older age, female sex, history of smoking, histological type associated with CIP, previous lung disease, prior thoracic irradiation, and treatment combinations with other drugs. Patients with these risk factors should be monitored for CIP when using ICIs. In terms of management, current guidelines have provided recommendations for CIP^113^. However, many questions remain, including screening biomarkers to predict the safety of ICIs, the relationship between the severity of adverse events and the effectiveness of immunotherapy, and the differences among diverse tumor histological types in CIP morbidity and mortality. These challenges need to be addressed in future clinical and preclinical studies. Moreover, because the current guidelines are based mainly on clinical experience and expert consensus, some recommendations remain controversial. Therefore, translation of preclinical data into clinical treatment and the development of guidelines supported by powerful evidence are essential.

## Figures and Tables

**Table 1:** Potential risk factors for checkpoint inhibitor pneumonitis in NSCLC

Risk factors	Details
Previous lung disease	COPD, asthma, ILD, pulmonary fibrosis, pneumothorax, and pleural effusion
Combination therapy	Additional immune drugs, targeted drugs, and chemotherapeutic drugs. ICI followed by osimertinib may be associated with severe pneumonitis. This association has not been observed when osimertinib preceded treatment with ICIs or when ICIs were followed by treatment with other EGFR-TKIs
Prior thoracic radiation therapy	The associations among chest-RT type, chest-RT timing, receipt of more than one chest-RT course, and CIP have not been proven
Smoking status	Previous or current smoker
Age	Older than 70 years
PD-1 inhibitors	PD-1 inhibitors, such as pembrolizumab and nivolumab, might be associated with a higher incidence of CIP than other ICIs
Different histological type of NSCLC	Patients with squamous NSCLC have a higher incidence but a lower mortality of CIP than those with adenocarcinoma

NSCLC, non-small cell lung cancer; COPD, chronic obstructive pulmonary disease; ILD, interstitial lung disease; ICIs, immune checkpoint inhibitors; TKIs: tyrosine kinase inhibitors; RT: radiation therapy; PD-1: programmed cell death protein 1.

**Table 2:** Incidence of checkpoint inhibitor pneumonitis in NSCLC patients treated with ICIs and other drugs

ClinicalTrials.gov identifier	Source	Phase	Histological types	Interventions	No. of patients	
					All-grade pneumonitis (%)	Grade ≥ 3 pneumonitis (%)
NCT02477826	CheckMate 227^70^	3	NSCLC	Arm I: nivolumab plus ipilimumab	Arm I: 4%	Arm I: 2%
				Arm II: nivolumab plus chemotherapy	Arm II: 2%	Arm II: 2%
				Arm III: chemotherapy	Arm III: 1%	Arm III:<1%
NCT02659059	CheckMate 568^72^	2	NSCLC	Nivolumab plus ipilimumab	6.9%	2.1%
NCT01454102						
	CheckMate 012^71^	1	NSCLC	Arm I: nivolumab 3 mg/kg every 2 weeks plus ipilimumab		
1 mg/kg every 12 weeks	Arm I: 5%	Arm I: 5%				
				Arm II: nivolumab 3 mg/kg every 2 weeks plus ipilimumab		
1 mg/kg every 6 weeks	Arm II: 3%	Arm II: 3%				
NCT02039674	KEYNOTE-021^73^	1/2	NSCLC	Arm I: pembrolizumab plus erlotinib	Arm I: 0%	Arm I: 0%
				Arm II: pembrolizumab plus gefitinib	Arm II: 14.3%	Arm II: 0%
NCT02454933	CAURAL^74^	3	NSCLC	Arm I: osimertinib plus durvalumab	Arm I: 17%	Arm I: 0%
				Arm II: osimertinib	Arm II: 18%	Arm II: 12%
NCT02143466	TATTON^66^	1b	NSCLC	Arm I: durvalumab	Arm I: 2.0%	Arm I: 0.6%
				Arm II: osimertinib plus durvalumab	Arm II: 38%	Arm II: 15%
NCT02578680	KEYNOTE-189^75^	3	Non-squamous	Arm I: pembrolizumab plus chemotherapy	Arm I: 4.4%	Arm I: 2.7%
				Arm II: placebo plus chemotherapy	Arm II: 2.5%	Arm II: 2.0%
NCT02366143	IMpower150^76^	3	Non-squamous	Arm I: bevacizumab plus chemotherapy	Arm I: 1.3%	Arm I: 0.5%
				Arm II: atezolizumab plus bevacizumab plus chemotherapy	Arm II: 2.8%	Arm II: 1.5%
NCT02039674	KEYNOTE-021^77^	2	Non-squamous	Arm I: pembrolizumab plus chemotherapy	Arm I: 7%	Arm I: 2%
				Arm II: chemotherapy	Arm II: 0%	Arm II: 0%
NCT02775435	KEYNOTE-407^65^	3	Squamous	Arm I: pembrolizumab plus chemotherapy	Arm I: 6.5%	Arm I: 2.5%
				Arm II: chemotherapy	Arm II: 2.1%	Arm II: 1.1%
NCT02367794	IMpower131^78^	3	Squamous	Arm I: atezolizumab plus chemotherapy	Arm I: 7%	Arm I: 1%
				Arm II: chemotherapy	Arm II: 1%	Arm II: 1%

NSCLC, non-small lung cancer; ICIs: immune checkpoint inhibitors.

**Table 3:** The prevalence of patients with potential risk factors for checkpoint inhibitor pneumonitis

Trial/author	Phase/real world	Immune checkpoint inhibitor	Risk factor	The incidence of any-grade pneumonitis	
				With risk factor (%)	Without risk factor (%)
Keynote-001^40^	Phase 1	Pembrolizumab	Asthma or COPD	5.4	3.1
Galant-Swafford et al.^41^	Real world	Mainly nivolumab or pembrolizumab	Asthma	11.5	4.3
Kanai et al.^45^	Real world	Nivolumab	ILD	31	12
Shibaki et al.^82^	Real world	Nivolumab or pembrolizumab	ILD	29	10
Yamaguchi et al.^46^	Real world	Nivolumab or pembrolizumab	Pulmonary fibrosis	35.1	5.8
Keynote-001^55^	Phase 1	Pembrolizumab	Thoracic radiotherapy	13	1
Voong et al.^58^	Real world	Mainly nivolumab or pembrolizumab	Thoracic radiotherapy	19	19
Keynote-407^65^	Phase 3	Pembrolizumab	Combination with chemotherapy	6.5	2.1
TATTON^66^	Phase 1b	Durvalumab	Combination with osimertinib	38	2.9
CAURAL^74^	Phase 3	Durvalumab	Combination with osimertinib	17	18
Oshima et al.^67^	Real world	Nivolumab	Combination with targeted TKI	25.7	4.6
Checkmate 227^70^	Phase 3	Nivolumab plus pembrolizumab	Double-immune checkpoint inhibitors	4	1

COPD, chronic obstructive pulmonary disease; ILD, interstitial lung disease; TKI, tyrosine kinase inhibitor.
